# Supplementary material for: Summer decapod crustacean larval communities along the eastern Spanish Mediterranean coast
Source: PLoS One. 2022 Nov 17;17(11):e0275892. doi: 10.1371/journal.pone.0275892 (PMC9671449; doi:10.1371/journal.pone.0275892)
Supplement: S1 Table — Lat.: Latitude; Long.: Longitude. Depth corresponds to bottom depth. (DOCX) [file pone.0275892.s001.docx]

S1 Table. Information of sampled stations. Lat.: Latitude; Long.: Longitude. Depth corresponds to bottom depth.

| **Date** | **Lat. (N)** | **Long. (E)** | **Depth (m)** |  | **Date** | **Lat. (N)** | **Long. (E)** | **Depth (m)** |
| --- | --- | --- | --- | --- | --- | --- | --- | --- |
| 22/07/2016 | -0.7242 | 37.4690 | 1006 |  | 16/08/2016 | 2.5473 | 41.1702 | 1038 |
| 22/07/2016 | -0.7508 | 37.5445 | 650 |  | 17/08/2016 | 2.5735 | 41.3575 | 248 |
| 25/07/2016 | -0.1022 | 37.6730 | 1304 |  | 17/08/2016 | 2.7615 | 41.4473 | 193 |
| 25/07/2016 | -0.1007 | 37.7198 | 591 |  | 17/08/2016 | 2.7897 | 41.4012 | 345 |
| 25/07/2016 | -0.4563 | 37.7130 | 144 |  | 17/08/2016 | 2.8272 | 41.3252 | 347 |
| 25/07/2016 | -0.3583 | 37.8850 | 184 |  | 17/08/2016 | 2.8557 | 41.2375 | 1154 |
| 26/07/2016 | -0.1283 | 37.8327 | 468.7 |  | 18/08/2016 | 3.0187 | 41.2557 | 1473 |
| 26/07/2016 | -0.1215 | 38.0868 | 387 |  | 18/08/2016 | 3.0055 | 41.3367 | 836 |
| 27/07/2016 | 0.0520 | 38.0283 | 1036 |  | 18/08/2016 | 3.5032 | 41.4412 | 1380 |
| 27/07/2016 | 0.4682 | 38.0730 | 1094 |  | 19/08/2016 | 3.2383 | 41.2838 | 1701 |
| 28/07/2016 | 0.3068 | 38.3490 | 204 |  | 19/08/2016 | 3.2242 | 41.3720 | 895 |
| 28/07/2016 | 0.4117 | 38.2037 | 884 |  | 19/08/2016 | 3.1165 | 41.4957 | 331 |
| 28/07/2016 | 0.5913 | 38.2137 | 853 |  | 20/08/2016 | 3.2918 | 41.5022 | 392 |
| 30/07/2016 | 0.6850 | 38.3448 | 904 |  | 20/08/2016 | 2.9417 | 41.5080 | 507 |
| 31/07/2016 | 0.7957 | 38.4833 | 884 |  | 19/08/2016 | 3.1558 | 41.4228 | 879 |
| 01/08/2016 | 0.6248 | 38.4748 | 709 |  | 19/08/2016 | 2.9698 | 41.4127 | 642 |
| 03/08/2016 | 0.5290 | 38.6670 | 365 |  | 22/08/2016 | 2.8203 | 41.5606 | 378 |
| 03/08/2016 | 0.7437 | 38.6760 | 848 |  | 23/08/2016 | 2.8917 | 41.6367 | 309 |
| 03/08/2016 | 0.9008 | 38.6558 | 528 |  | 23/08/2016 | 2.8547 | 41.6290 | 201 |
| 03/08/2016 | 1.0715 | 38.7228 | 120 |  | 23/08/2016 | 2.8850 | 41.5683 | 93 |
| 04/08/2016 | 0.9537 | 38.8165 | 533 |  | 23/08/2016 | 3.1013 | 41.5620 | 133 |
| 04/08/2016 | 0.7418 | 38.7303 | 855 |  | 23/08/2016 | 3.3795 | 41.7067 | 187 |
| 04/08/2016 | 0.4238 | 38.8088 | 138 |  | 23/08/2016 | 3.4030 | 41.6697 | 288 |
| 04/08/2016 | 0.3933 | 38.9420 | 249 |  | 23/08/2016 | 3.4342 | 41.5820 | 827 |
| 04/08/2016 | 0.5502 | 38.9512 | 776 |  | 23/08/2016 | 3.4987 | 41.5093 | 1797 |
| 04/08/2016 | 0.7158 | 38.9593 | 857.7 |  | 24/08/2016 | 3.6378 | 41.5997 | 1206 |
| 05/08/2016 | 0.9092 | 38.9843 | 728 |  | 24/08/2016 | 3.3415 | 41.8232 | 174 |
| 05/08/2016 | 0.8358 | 39.1272 | 1082 |  | 25/08/2016 | 3.2717 | 41.8610 | 129 |
| 06/08/2016 | 0.6960 | 39.0325 | 1030 |  | 25/08/2016 | 3.3538 | 41.9002 | 298 |
| 07/08/2016 | 0.4812 | 39.0395 | 916 |  | 25/08/2016 | 3.4140 | 41.8922 | 515 |
| 07/08/2016 | 0.2795 | 39.0735 | 328 |  | 25/08/2016 | 3.5862 | 41.6697 | 704 |
| 07/08/2016 | 0.5355 | 39.2563 | 1295 |  | 26/08/2016 | 3.5483 | 41.7433 | 1110 |
| 08/08/2016 | 0.4728 | 39.5425 | 876 |  | 26/08/2016 | 3.5070 | 41.8170 | 1840 |
| 08/08/2016 | 0.6273 | 39.4728 | 1161 |  | 26/08/2016 | 3.4410 | 41.8887 | 591 |
| 09/08/2016 | 0.7535 | 39.5622 | 1104 |  | 26/08/2016 | 3.4248 | 41.9788 | 187 |
| 09/08/2016 | 0.8123 | 39.7935 | 526 |  | 26/08/2016 | 3.3948 | 42.0592 | 130 |
| 10/08/2016 | 0.3165 | 39.7080 | 116 |  | 27/08/2016 | 3.5191 | 42.1618 | 250 |
| 10/08/2016 | 0.9843 | 40.1660 | 90 |  | 27/08/2016 | 3.4609 | 42.2327 | 123 |
| 10/08/2016 | 1.3548 | 40.1937 | 1084 |  | 27/08/2016 | 3.3855 | 42.3362 | 462 |
| 11/08/2016 | 1.2870 | 39.9563 | 1355 |  | 27/08/2016 | 3.3187 | 42.3753 | 113 |
| 11/08/2016 | 1.2093 | 40.0995 | 822 |  | 27/08/2016 | 3.3911 | 42.3755 | 366 |
| 11/08/2016 | 1.2903 | 40.3365 | 126 |  | 27/08/2016 | 3.4520 | 42.3692 | 200 |
| 12/08/2016 | 1.4685 | 40.3492 | 1341 |  | 27/08/2016 | 3.5317 | 42.3437 | 711 |
| 14/08/2016 | 1.3070 | 40.6348 | 115 |  | 27/08/2016 | 3.7430 | 42.4253 | 844 |
| 14/08/2016 | 1.4862 | 40.6578 | 940 |  | 28/08/2016 | 3.8007 | 42.2844 | 995 |
| 14/08/2016 | 1.5415 | 40.8740 | 680 |  | 28/08/2016 | 3.6697 | 42.1917 | 533 |
| 15/08/2016 | 1.3173 | 40.9607 | 147 |  | 28/08/2016 | 3.7298 | 42.0465 | 951 |
| 15/08/2016 | 1.3945 | 40.7777 | 404 |  | 28/08/2016 | 3.5992 | 41.9573 | 854 |
| 15/08/2016 | 1.8020 | 41.1183 | 82.6 |  | 28/08/2016 | 3.6878 | 41.8280 | 1626 |
| 15/08/2016 | 1.8895 | 40.9580 | 1143 |  |  |  |  |  |
| 16/08/2016 | 2.1730 | 41.1395 | 432 |  |  |  |  |  |
| 16/08/2016 | 2.5133 | 41.2980 | 695 |  |  |  |  |  |
